# Cure of Tubercular Consumption and Scrofula

**Published:** 1835

**Authors:** 


### IV. Cure of tubercular Consumption and Scrophula.

We give the following extract of a letter from Dr. H. H. Sherwood, formerly of the state of New-York, now a resident of Hamilton, in this state, with the single remark, that he is a Physician of regular education.

"I have been investigating the subject of Tuburcula, or what is called Scrofula, for more than a quarter of a century, and in 1812 found new and unerring symptoms by which I could detect the disease in any part of the system, and also found remedies that cure all the cases in every part of the system and in all its stages, with a few exceptions of extensive disorganization and loss of substance.

All the cases with these exceptions, commence getting well in a few hours after the patients begin the use of the remedies, and this operation continues and cannot easily be diverted from its course until the disease is cured, and with a few exceptions they are all cured in from three to seven weeks. It never produces any disagreeable or injurious effects upon the stomach or bowels, or any other part of the system and dieting never necessary or proper.

I have now used these remedies for the cure of this disease in every part of the system; and have gradually increased their power until they have now, and have had, for a number of years past, all the efficiency that can be expected or perhaps desired; and having with many others satisfied myself that there cannot possibly be any mistake, either in the symptoms in any part of the system or in the remedies for its cure, I am anxious to extend their benefits as quickly and as widely as possible, for the purpose of lessening the great loss of human life by this disease.

In investigating this disease by the symptoms, which unerringly point to it in every part of the system, it will not surprise you to hear, that there are many diseases in the different parts of the system that are really, but rarely if ever suspected to be tubercular, and aware of your exertions, experience, and success in extending the benefits of the science of medicine, I am anxious to avail myself of your advice as regards the best and most proper manner of communicating to the profession its true phenomena, symptoms and remedies.

I have occupied my leisure hours this winter, in writing a short description and history of the disease, with a description and cause of their new symptoms; and besides have copied nearly fifty cases affecting different parts of the system, with a view of publishing a small work of 150 or 200 pages duodecimo, which will be ready for the press in two or three weeks, and as it is not necessary to my purpose to wade in the labarinths of supposition, it appears to me that a work of a larger size would be unnecessary."

